# Gender and Monitoring the Response to HIV/AIDS Pandemic^1^

**DOI:** 10.3201/eid1011.040498

**Published:** 2004-11

**Authors:** Paul DeLay

**Affiliations:** * Joint United Nations Programme on HIV/AIDS, Geneva, Switzerland

**Keywords:** epidemiologic monitoring, HIV, acquired immune deficiency syndrome, AIDS, perspective

## Abstract

The mechanisms, techniques, and data sources used to monitor and evaluate global AIDS prevention and treatment services may vary according to gender. The Joint United Nations Programme on HIV/AIDS has been charged with tracking the response to the pandemic by using a set of indicators developed as part of the Declaration of Commitment endorsed at the U.N. General Assembly Special Session on AIDS in 2001. Statistics on prevalence and incidence indicate that the pandemic has increasingly affected women during the past decade. Women's biologic, cultural, economic, and social status can increase their likelihood of becoming infected with HIV.

## Abstract 2

Statistics on prevalence and incidence indicate that the HIV/AIDS pandemic has increasingly affected women during the past decade.

Since 2000, global financial resources have increased to allow expansion of both prevention and treatment services through a number of new initiatives, such as the Global Fund to Fight AIDS, TB and Malaria; the U.S. President's Emergency Plan for AIDS Relief; and the World Bank MAP program. Programs should be monitored and evaluated to ensure these investments are used to maximum effect. Different types of data should be included when assessing the status of the HIV/AIDS epidemic and effectiveness of the response. Each of these "data streams" provides information to enhance program planning and implementation.

## Types of Data

### Biologic Surveillance

These types of data include the prevalence of HIV infection in the general adult population and in persons at high risk, as well as the actual estimated number of persons who are infected, who have symptoms of AIDS, and who died of the disease. These data are generally obtained from a combination of sentinel surveillance methods and biologic testing as part of community population surveys.

### Policy Environment

These types of data include establishing policies that increase access to services, protect the rights of vulnerable groups, and provide adequate resources. These data are primarily obtained from a set of discrete tools that include the National Composite Index and the AIDS Programme Effort Index; both tools review legislation, policies, and key interviews of relevant groups, including government officials, clinical providers, and persons affected by or living with HIV/AIDS.

### Behavioral Surveillance

These types of data measure levels of risk for HIV transmission and changes in risk levels over time. Major population survey questionnaires, such as demographic health surveys, gather this information, along with targeted behavioral surveillance, such as the Behavioral Sentinel Surveillance Surveys, pioneered by Family Health International.

### Resource Flows Data

These types of data include tracking the contributions from external donors (bilateral, World Bank, Global Fund to Fight AIDS, TB and Malaria; and international foundations) as well as national expenditures and "out of pocket" spending from families and persons affected by HIV/AIDS. These data are generally obtained from global resource tracking databases (e.g., Organization for Economic Cooperation and Development) and at the country level from subanalyses of National Health Accounts and through the use of a new tool, National AIDS Accounts.

### Tracking Commodities

These types of data provide useful proxy information on program implementation. They are often only available from the donor community that procures these commodities (drugs, condoms, and HIV diagnostic kits) and are rarely routinely collected as part of a country's health information system.

### Prevention and Treatment Services

These types of data are generally measured through health information systems. However, because such systems are often inadequate in many developing counties, these data remain poor. Tracking services, for prevention or treatment, also necessitates monitoring the quality of these services. Much of this information can be obtained from health facility surveys, but these surveys are staff- and resource-intensive and are generally not conducted routinely.

### Disease and Death Data

These types of data are frequently obtained from vital events registration, including estimated births, deaths, and causes of death. Again, in many developing countries, the collection of this type of information tends to be deficient.

### Response Indicators

The Joint United Nations Programme on HIV/AIDS (UNAIDS) has been charged with tracking the response to the pandemic by using a set of indicators developed as part of the Declaration of Commitment, which was endorsed at the U.N. General Assembly Special Session on AIDS in 2001. When these indicators were first measured in 2003, differences in the comprehensiveness and quality of available data became clear. The biologic surveillance data are improving over time, and now the policy environment can be assessed to determine levels of commitment. However, we currently obtain very poor data on commodities, resource flows, and the coverage and quality of prevention and treatment services. This lack of credible data is made worse by the inability to disaggregate these activities by gender.

## Biologic Surveillance Data

**Figure 1:**
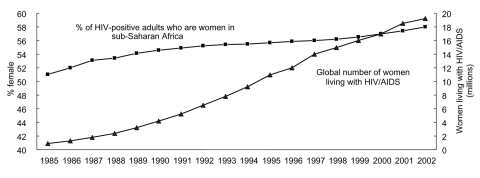
Feminization of HIV/AIDS epidemic, 1985–2002. Source: United Nations Joint Programme on HIV/AIDS, World Health Organization. Estimates; 2002.

Until recently, most of this information has been gathered from sentinel surveys of general adult populations and populations at risk. These data are now being complemented by the increasing use of population surveys that include seroprevalence testing. These new data collection methods deepen our understanding of the pandemic. Statistics on prevalence, incidence, and estimated numbers of persons infected with HIV and those with HIV disease have gender implications and point to the increased feminization of the pandemic over the past decade. Almost 60% of HIV-infected persons in sub-Saharan Africa are female. Nearly 20 million HIV-infected persons in the developing world are girls and women (Figure 1). Biologic and behavioral data explain why these different vulnerabilities exist and what sort of interventions could address these different transmission dynamics. Other data indicate that women in sub-Saharan Africa are often infected in their teens while men become infected in their 20s, a 10-year difference.

### Policy Environment

A major focus of The U.N. General Assembly Declaration of Commitment is to examine whether countries themselves are creating an environment with a positive and effective response. Are the appropriate policies in place? Is there real commitment by government and civil society to implementing programs that will make a difference? UNAIDS attempts to examine stigma and discrimination by using the National Composite Policy Index (NCI), the AIDS Program Index (API), and a series of media analyses. Globally, funds made available by various sources, HIV/AIDS policies within multinational corporations, and the value of global advocacy efforts are measured. At the national level, funds from national governments, existence of policies to promote programs for risk reduction, and access to care and treatment are measured.

NCI assesses legislation and policy related to national strategic plans, prevention efforts, maintenance of human rights, and provision of care and support. For instance, if a national strategy exists for HIV/AIDS, does it address those at risk in the uniformed services, youth, migrants, and other risk groups? Does the strategy address human rights, particularly access to services for women? However, this approach does not assess the public's awareness of this legislation, how the policy is enforced and implemented, or the quality of these policies. For example, in sub-Saharan Africa, 80% of developing countries have a policy in place to provide antiretroviral therapy, but only 1% of HIV-infected persons in this region actually have access to these lifesaving treatments.

In addition to NCI, which quantitatively measures the policy environment, we are also using API, which is a more qualitative, subjective index that examines a range of issues, including stigma, discrimination, and access to services. API is measured by using a Delphi approach. Relevant groups (civil society, health workers, policymakers, government officials, businessmen, religious leaders, persons living with AIDS) are brought together and asked a series of ≈100 standardized questions. This instrument allows us to assess whether policies are actually being implemented and whether they are making a difference. This tool has become increasingly useful to measure subtle and complex changes that occur in a country response, and it is now being applied to other interventions, such as the scope and quality of orphan care, which is difficult to track quantitatively.

One downside to the API is that it does not allow for cross-country comparison. The reason is that citizens' expectations about their government vary widely across countries. Some countries may be assessed poorly because the citizens expect more; however, a country with no policy may do well because the expectations from the public are much lower. However, API is being revised to make some of the responses to the structured interview questions less subjective.

### Behavioral Risk Data

Behavioral data fall into three areas, which include data on vulnerability, knowledge and awareness, and risk behavior over time. Women have biologic, cultural, economic, and social vulnerabilities that increase their likelihood of acquiring an infectious disease. They also face hostile justice systems, are exposed to sexual violence, lose inheritance rights and property, lack access to reproductive services, and have few support systems

Analysis of data can lead to a better understanding of inherent vulnerabilities. For example, marriage does not necessarily protect against sexually transmitted infections. Marriage at a very young age can actually increase vulnerability, initiating a young woman into sexual activity earlier than if she were not married. While we believe these findings could be a pathway for future potential interventions, exactly what these programs would look like is being debated.

**Figure 2:**
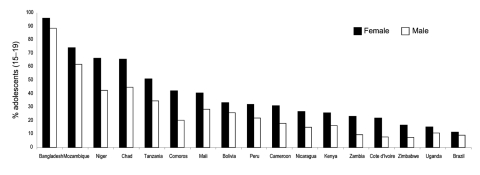
Percentage of adolescents (15–19 years) who do not know how to protect themselves from HIV. Source: Macro International, USA and United Nations Children's Fund, demographic and health surveys.

Figure 2 presents a collection of the knowledge and awareness data that are now being measured in many countries on a more routine basis. Data on the knowledge of how a person becomes infected and how we can protect ourselves are often better disaggregated by gender than many of the other data streams because knowledge assessment surveys are often performed in a same-sex venue. In many ways, girls understand prevention data better than boys. Girls tend to be more accessible to the transfer of information. However, their ability to act on this knowledge is often where the vulnerabilities lie.

**Figure 3:**
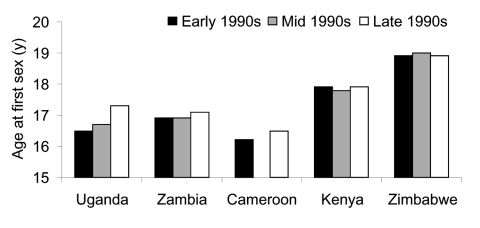
Median age at first sexual intercourse among young women. Source: World Health Organization. HIV/AIDS epidemiologic surveillance update for the WHO Africa region.

The age of first sexual activity can be closely correlated with HIV prevalence (Figure 3). However, the definition of sexual debut can be difficult because much of these data come from retrospective interviews. Persons are asked to remember when their first sex act occurred. The validity of some of these data is questioned, and these methods do not easily permit an assessment of current sexual practices of young persons.

Cross-generational sex is a controversial, but important, area when considering vulnerability. Recently, efforts have been increased to better understand what is happening in this area. While exploitation is a reality, not all persons involved perceive these acts as exploitation. Most young women participate in these relationships to survive. Older men think that they reduce their chance of being infected by being with a younger girl. Addressing these complex and culturally sensitive issues will require innovative thinking.

**Figure 4:**
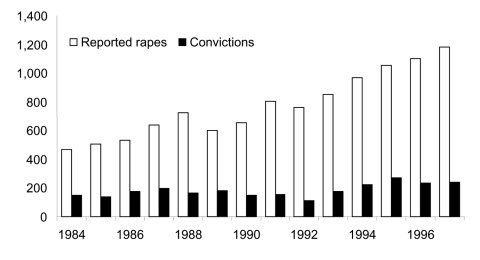
Number of reported rapes and convictions in Botswana. Source: Emang Basadi Women's Association, Botswana, 1998.

Violence is another major issue that makes women vulnerable. Data from South Africa indicate that 33% of women are afraid of saying no to sex, 29% have been forced to have sex, and 55% have had sex because their boyfriend insisted (*1*). In Botswana, the number of reported rapes has increased, but the number of convictions has not (Figure 4).

## Tracking Resource Flows

**Figure 5:**
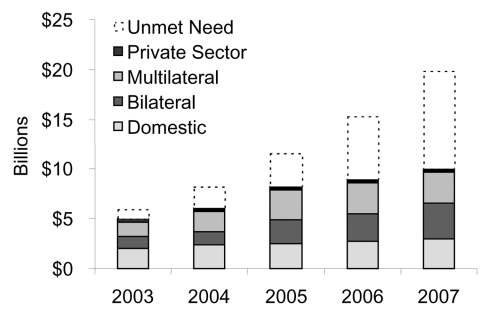
Projected International HIV/AIDS Resource Need and Funding Availability, 2002–2005. Source: United Nations Joint Programme on HIV/AIDS. Report on the state of HIV/AIDS financing; 2003.

While not traditionally part of monitoring and evaluation, tracking financial resource flows is now considered a key element for monitoring program implementation and effectiveness. How much money comes from the U.S. government or other international donors into a country versus how much comes from the national government itself? Do you see economies of scale as service coverage goes up? Do commodities, such as antiretroviral drugs, become more inexpensive, or as we move from first-line to second-line antiretroviral treatments, will the costs actually go up over time. At a global level, we rely on the Organization for Economic, Cooperation, and Development, which was created by the major industrialized countries to track financial resources and by the Netherlands Interdisciplinary Demographic Institute, which is under contract to UNAIDS to provide information on funding levels from bilateral donors, multilateral organizations, foundations, and the private sector. Resource flows are often tracked at the country level through National Health Accounts and National AIDS Accounts. Figure 5 shows funding levels for AIDS activities in the developing world from various sources.

While spending is increasing, so are unmet needs. The estimated need represented by the top portions of the bar chart (Figure 5) does not include the necessary investments in infrastructure, such as the costs for building more hospitals, clinics, and laboratories, and for training more people. These estimated needs represent what could be spent now with existing infrastructure. The right resources should go to the right places. What should the public sector pay for? What should the private corporate sector pay for? These ratios will change from country to country. We need to examine efficiency of allocations. Assessing equity of allocations is equally important. Is the money actually going to the populations that need it the most? Is it going to a general Information Education Communication campaign, or is it going to the populations at highest risk? Is it staying in the cities or reaching rural settings?

## Service Delivery and Coverage

To examine services and coverage, we use key informant coverage surveys, health facility surveys, and donor data. In the UNAIDS 2003 Progress Report on the Global Response to the HIV/AIDS Epidemic, the available data on the delivery of preventive and treatment services demonstrated a lack of access. Only 1 in 100 pregnant women receives services to prevent mother-to-child transmission of HIV. One in 100 eligible HIV-infected persons (men and women) has access to antiretroviral therapy. One in 10 has access to testing for HIV infection. One in four has access to basic information on AIDS and protection against AIDS. Much of these data cannot currently be disaggregated by gender. When we look at antiretroviral therapy, ≈400,000 persons in the developing world are being treated, of which ≈80,000 are in sub-Saharan Africa, but the ratio of women to men receiving these services is difficult to break down.

## Overall Challenges

We face a number of challenges in containing the AIDS pandemic and increasing prevention and treatment programs, particularly as they relate to gender. Setting standards and targets for equity of access and then monitoring progress will be critical. For example, UNAIDS advocates that of the 3 million infected persons who should be started on antiretroviral therapy by the end of 2005, an appropriate number should be women, depending on the ratio of infected men to women in that specific country.

Measuring sectorwide effects of these new resources will be a major issue. If a massive investment in infrastructure is focused on the three major infectious diseases (AIDS, tuberculosis, and malaria), will the health sector be positively affected as a whole or will we see staff, commodities, and resources diverted from other areas of public health, such as immunization of children or treatment of childhood diarrhea? How are we going to measure this?

A new initiative, the Global Coalition on Women and AIDS, was launched February 2004 by UNAIDS and other partners. The coalition focuses on selected key areas: preventing HIV infection in girls and women, reducing violence against women, protecting property and inheritance rights, ensuring equal access by women and girls to care and treatment, and supporting efforts for universal education for girls. We need to find ways to accurately measure progress in all these areas, while recognizing that quantitative methods may not be the most appropriate way to get a true picture of progress or lack thereof.

We are making progress in monitoring and evaluating prevention measures, but we have a long way to go. As we collect appropriate biologic, policy, behavioral, and service coverage data, patterns of gender dynamics and inequities begin to emerge. We must use this information to address these inequities.
